# Paleo-Immunology: Evidence Consistent with Insertion of a Primordial Herpes Virus-Like Element in the Origins of Acquired Immunity

**DOI:** 10.1371/journal.pone.0005778

**Published:** 2009-06-03

**Authors:** David H. Dreyfus

**Affiliations:** 1 Allergy and Clinical Immunology/Pediatrics, Yale School of Medicine New Haven, New Haven, Connecticut, United States of America; 2 Keren Pharmaceutical, New Haven, Connecticut, United States of America; National Institute on Aging, United States of America

## Abstract

**Background:**

The RAG encoded proteins, RAG-1 and RAG-2 regulate site-specific recombination events in somatic immune B- and T-lymphocytes to generate the acquired immune repertoire. Catalytic activities of the RAG proteins are related to the recombinase functions of a pre-existing mobile DNA element in the DDE recombinase/RNAse H family, sometimes termed the “RAG transposon”.

**Methodology/Principal Findings:**

Novel to this work is the suggestion that the DDE recombinase responsible for the origins of acquired immunity was encoded by a primordial herpes virus, rather than a “RAG transposon.” A subsequent “arms race” between immunity to herpes infection and the immune system obscured primary amino acid similarities between herpes and immune system proteins but preserved regulatory, structural and functional similarities between the respective recombinase proteins. In support of this hypothesis, evidence is reviewed from previous published data that a modern herpes virus protein family with properties of a viral recombinase is co-regulated with both RAG-1 and RAG-2 by closely linked cis-acting co-regulatory sequences. Structural and functional similarity is also reviewed between the putative herpes recombinase and both DDE site of the RAG-1 protein and another DDE/RNAse H family nuclease, the Argonaute protein component of RISC (RNA induced silencing complex).

**Conclusions/Significance:**

A “co-regulatory” model of the origins of V(D)J recombination and the acquired immune system can account for the observed linked genomic structure of RAG-1 and RAG-2 in non-vertebrate organisms such as the sea urchin that lack an acquired immune system and V(D)J recombination. Initially the regulated expression of a viral recombinase in immune cells may have been positively selected by its ability to stimulate innate immunity to herpes virus infection rather than V(D)J recombination Unlike the “RAG-transposon” hypothesis, the proposed model can be readily tested by comparative functional analysis of herpes virus replication and V(D)J recombination.

## Introduction

Biological systems can share a common mechanism either because of descent from a common ancestral system or molecule (termed homology), or because of convergent evolution of two unrelated systems or molecules (termed analogy). In contrast to non-biological systems, the previous history of a biological system is critical in understanding both the origins of the system and its functional properties. Distinction between homologous and analogous similarities is also useful in providing empirically testable hypothesis regarding the origins of complex biological systems such as the acquired immune system that originated in the distant past [1]–[7].

Soon after the mechanism of V(D)J recombination was discovered homology was evident between V(D)J recombination and the biology of mobile DNA sequences termed transposons [1], [2] as well as retroviral integration [8], [9]. RAG-1/RAG-2 protein complex required for recombination of genes for immunoglobulin and T cell receptor genes *in vivo* can function as a transposase under some conditions *in vitro* (although apparently not at a high rate *in vivo*) [10]–[13]. Importantly, however, the RAG-1 protein differs from existing transpose molecules such as the transposase of the “transib” transposon family due to the addition of an amino terminus that appears to be a member of another multi-gene protein family [6].

Possibilities include either a “big bang” simultaneous insertion of a transposon and origin of V(D)J recombination as proposed initially [1], [14], or a more gradual process [7]. Recent sequence data from the complete sea urchin genome demonstrates that the sea urchin seems to encode a functional RAG-1 protein adjacent to a gene encoding a functional RAG-2 protein although the sea urchin does not have any evidence of an acquired immune system, favoring a gradual rather than “big bang” model [7]. However, if the origins of the acquired immune system was a gradual process rather than a “big bang” there is no explanation for the selective pressure favoring maintenance and expression of the functional RAG-1/RAG-2 like locus for long periods of time until the present in the sea urchin in the absence of V(D)J recombination.

Another recent observation bearing on the origins of acquired immunity is that co-localization and co-regulation of RAG-1 and RAG-2 occurred prior to the origins of V(D)J recombination but after fusion of a transib like element with an unrelated amino-terminal protein [6]. Since there are no known transposable elements that encode a transposase intermediate between the transib transpose and RAG-1 with respect to the amino terminus section of RAG-1, this would imply that another intermediate form of mobile element with an amino terminus similar to RAG-1 preceded the insertion of the RAG-1 precursor gene adjacent to the RAG-2 gene. However, no such mobile element has been detected despite complete sequencing of the human and other vertebrate genomes and extensive, focused analysis of these sequences [6], [7].

As shown in this work, these limitations of the transposon insertion theory of the origins of acquired immunity can be eliminated with a radically different model of the origins of the acquired immune system. In this model infection of the germ line of a primordial or ancestral deuterostome with a primordial herpes virus in the distant past, prior to the origins of acquired immunity led to capture of this auto-regulatory episome by the vertebrate germ line genome adjacent to a primordial RAG-2 like gene. In a single event, an episomal encoded recombinase resembling a primordial RAG-1-like gene then could then co-regulate and co-evolve with RAG-2 in somatic tissues such as lymphocytes.

Most importantly, a selective advantage to this event may have resulted and persisted until the present not from the recombinogenic properties of the new gene locus, but rather only a selective advantage due to expression of an antigenic protein from a herpes virus in the immune system of the organism. Expression of this antigenic herpes virus protein could have provided a selective advantage to descendent of the organism containing the inserted pathogen through interactions with somatic elements of the inate immune system contributing to enhanced innate immunity to herpes infection. In this model, RAG-2, rather than stimulating or regulating V(D)J recombination was initially a repressor of recombination preventing adverse unregulated recombination events with existing transib-like elements consistent with evidence that RAG-2 may under some conditions block rather than augment RAG-1 function [15], [16].

The herpes virus replication cycle has some functional similarity to the excision and recombination of V(D)J episomes during the generation of the T and B cell repertoire although whether this is an analogous or homologous process is not known. Epstein-Barr Virus (EBV, also denoted human herpes virus 4) infection results in activation of the RAG genes required for V(D)J recombination [17]–[20], suggesting that the viral and host genes are part of a similar regulatory network. A conserved family of herpes virus DNA binding proteins (denoted BALF-2 protein in EBV and ICP-8 in Herpes Simplex) is required for viral replication, which involves a complex series of recombination events [21], [22]. Representative members of this protein family from widely divergent herpes proteins have similar biochemical properties to the RAG-1 protein suggesting that herpes virus replication and V(D)J recombination could be related through a process of homologous adaptation from a precursor recombinase in the DDE/RNAse H family of enzymes [17], [23]. Two novel empirical observations are discussed in connection with this model of the acquisition of acquired immunity.

First, it is shown that the cis-acting regulatory sequences required for co-regulation of a putative primordial RAG-1/RAG-2 recombinase are functionally similar to cis-acting sequences regulating the current herpes recombinase protein termed the herpes major DNA binding protein (DBP) accounting in part for the previous regulatory interactions between the RAG proteins and herpes virus infection of lymphocytes [17], [23] Second, it is shown that the recently solved partial crystal structure of a conserved herpes virus recombinase, the DBP protein ICP-8 [21], [22] shares functional properties with the known structural features of both the RAG-1 recombinase and RISC (RNA induced silencing complex). RISC, a vertebrate member of the DDE/RNAse H family of enzymes whose structure has been solved completely binds and cleaves single and double stranded nucleic acids, although apparently restricted to substrates of RNA rather than DNA [24], [25]. RAG-1 and RISC both utilize magnesium ions in a site functionally related to the DDE site of RNAse H, also shared with retroviral integrases [25], [26]. Like RISC, and RAG-1, ICP-8 has a magnesium ion dependent strand exchange function and exhibits conformation changes in the presence of Mg++ [21].

Similar interactions between modern-day RISC, RAG and modern-day herpes recombinases, and substrates such as nucleic acids may result as a vestige of a shared recombination mechanism and evolutionary history, a form of molecular “arms race” between recombinases shared between both infectious agents and the acquired immune system. As a consequence of this immunologic “arms race,” primary sequences similarities between the herpes recombinases and the RAG proteins have been selected against and thus are not readily evident, while functional similarities in the regulation, structure, and function of the respective recombinases have been relatively preserved. The importance of these observations is that certain empirically testable predictions can be made regarding other functional and regulatory properties of the herpes DBP and herpes recombination shared with the acquired immune system. Conversely, since the “rag transposon” has never been observed experimentally it cannot be assumed to exist, and should not be cited uncritically in discussions of the origins of the acquired immune system.

## Results

### Identification of sequences resembling V(D)J RSS and transposon termini in the termini of EBV and Herpes Simplex

**Figure 1:**
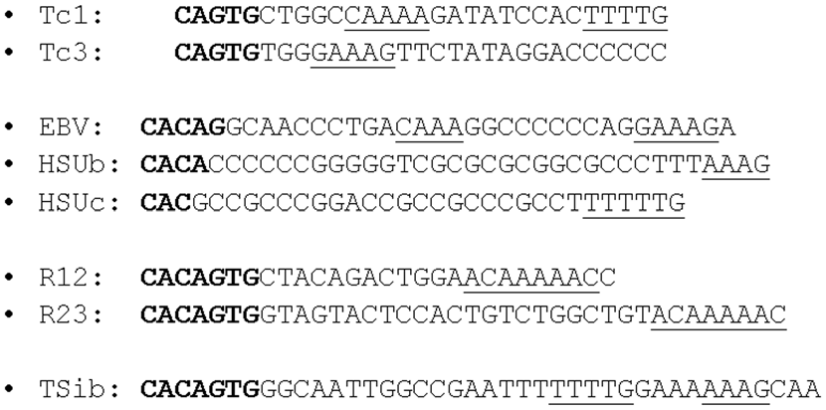
Mechanism of Tc element transposition, herpes recombination and V(D)J recombination suggests homologous adaptation of a primordial DDE recombinase. Recombination sites of all of these DNA elements are adjacent to a sequence containing a sequence resembling the V(D)J heptamer (bold type) and nonamer sequences (underlined). Nonamer sequences are often spaced at 12 or 23 nucleotide intervals to facilitate DNA bending. Sequences shown from top to bottom: invertebrate Tc elements Tc1, Tc3, EBV terminal repeat sequences, herpes simplex recombination sites, V(D)J RSS and transib transposon termini (Tsib) most closely related to V(D)J RSS among tranposons.

**Figure 2:**
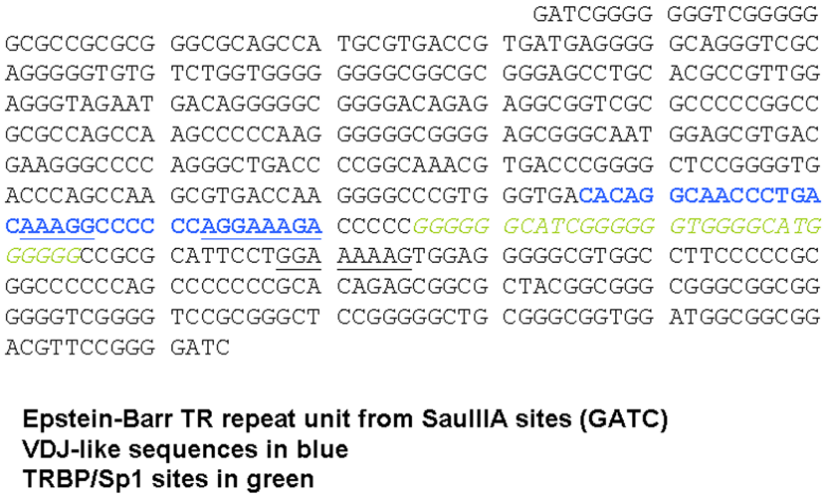
The complete sequence of an EBV terminal repeat defined by the SauIII restriction enzyme is shown with putative V(D)J-like regions and Sp1 transcription factor binding sites identified. Location of V(D)J-like sequences in EBV terminal repeats adjacent to experimentally confirmed Sp1 protein binding sites. Specific protein complexes distinct from Sp1 are also evident on the EBV V(D)J-like sequences shown , and these sequences undergo anomalous migration on native polyacrylamide gels typical of bent DNA similar to the V(D)J RSS and to transposon termini (unpublished observations).

Sequences in the EBV genome were first examined to determine whether regions of the genome undergoing recombination during viral replication resembled V(D)J recombination signals (whole EBV genome analysis not shown). The genome and termini of EBV have enriched G/C content (approximately 70% G/C), thus regions resembling a V(D)J RSS nanomer (A/T rich regions that contribute to DNA bending and interactions with bending proteins) are relatively uncommon. Only three potential nonomer-like sequences occur in the EBV terminal repeats which undergo a high rate of deletion and duplication during viral replication.. Only one of these nonomer-like sequences in the EBV TR is adjacent 5′ to a sequence with any similarity to V(D)J RSS and transposon termini. This sequence is shown in comparison to V(D)J RSS and transposon termini in Figure 1. The location of these sequences is shown within the complete EBV terminal repeat unit sequence defined by SauIII restriction enzyme sites in Figure 2.

A similar process was used to identify V(D)J RSS-like regions shown in Figure 1 occurring in published regions of herpes simplex that, like the TR of EBV, undergo complex inversions during viral replication [27] (Figure 1)). EBV terminal sequences shown in Figures 1 and 2 undergo anomalous migration in polyacrylamide gels due to phased poly-A tracts spaced 12 nucleotides along the alpha helix (unpublished observations). Recombination events resulting in contraction and expansion of the number of EBV terminal repeats (TR) are apparently localized to the TR element shown in Figure 2, since each packaged viral genome has only a single repeat element while the episomes generated during initial lymphocyte infection have multiple copies of the repeat [28], [29]. The virus undergoes a high rate of other rearrangements during viral replication suggesting activation of a specific recombination pathway [30].

### The “RAG-transposon insertion model” of the origins of V(D)J recombination: Do the ends justify the means?

Like DNA transposons, herpes viruses are DNA mobile sequences (moving horizontally among somatic cells through an infectious process) rather than vertically through transposition in the genome. Herpes viruses are capable of both closed circular episome formation [29] and linear insertion into host genomes, and infection of lymphocytes causes activation of RAG expression [17], [19], [31]. These observations (Figure 1) led the author more than a decade ago to propose that herpes viruses might encode a recombinase similar to the DNA transposases and RAG-1 protein [17].

A conserved family of herpes virus DNA binding proteins (DBP denoted BALF-2 protein in EBV and ICP-8 in Herpes Simplex) are required for viral replication which involves a complex series of recombination events [21], [22]. The herpes DBP ICP-8 from herpes simplex co-precipitates with proteins such as Ku and DNA pK [32] that are functionally associated with RAG-1 protein during V(D)J recombination, and additional structural information has become available regarding the molecular structure of the herpes simplex DBP [21], [22]. The remainder of this work will elaborate a novel hypothesis , namely that a primordial herpes virus, rather than a transib or similar DNA transposon was the source of the primordial RAG-1 protein, and that understanding the biology of herpes virus replication is therefore essential to understanding the origins of the acquired immune system.

Herpes viruses undergo a lifecycle characterized by circularization of a large infectious DNA linear form in somatic cells [33]. The circular somatic episome persists in a latent form in the latently infected cell until a signal triggers a program of lytic gene expression such as transcription of the viral transcription factor BZLF-1 [34], [35], activating other viral and host genes and causing the circular episome to linearize and form infectious viral particles. For example EBV exists in a latent state solely in B- lymphocytes, but can also transiently infect and in some cases cause malignant transformation of T-lymphocytes [17] or epithelial cells [36].

Comparison of herpes virus terminal sequences to transposon termini and V(D)J RSS suggests that these recombination pathways are homologous (rather than analogous) (Figure 1) [23]. In EBV replication, representative of the herpes replication cycle, sequences resembling V(D)J RSS in a repeated array containing variable numbers of a repeat element. Variation in the copy number of the terminal repeat elements may play a role in viral gene expression and binding of transcription factors such as Sp1 (Figure 2). The Sp1 transcription factor binds immediately adjacent to the cleavage site in the terminal repeat element generating the linear viral form [37], [38].

### The RAG-2 problem

**Figure 3:**
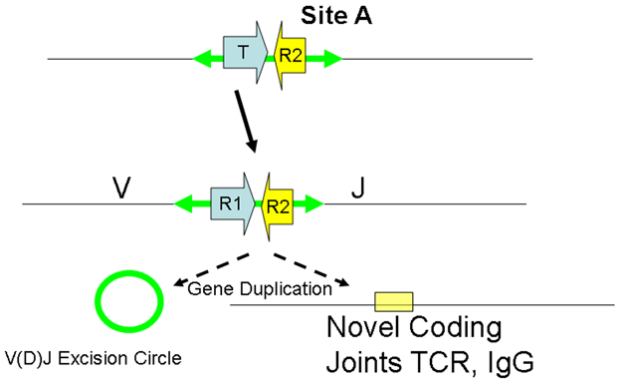
The “big bang” or “RAG-transposon insertion model” of the origins of the acquired immune system. As shown , a transposon inserted at a site in the genome denoted Site A can transcribe an mRNA encoding a bi-molecular transposase consisting of RAG-1 and RAG-2 like proteins from promoters in flanking sequences. Expression of the transposase then can excise a transposon from site A or another site (large arrows) at the transposon termini and insert the transposon and another site termed site B with an immunoglobulin or T-cell receptor gene. Subsequent excision of V(D)J RSS that resemble transposon termini results in circular episomes and repaired empty sites in immunoglobulin and T cell receptor genes. Multiple cycles of RAG transposon insertion and excision from primordial immunoglobulin and T-cell receptor like genes and insertion of the RAG transposon at other sites in the genome such as the current RAG locus with subsequent gene amplification of the immunoglobulin and T cell receptor gene families could result in the current structure of these genetic loci.

As shown in Figure 3, current model of the origins of V(D)J recombination proposes that a mobile element termed a “RAG transposon” contained both V(D)J like terminal sequences as well as a functional transposase or transposase complex. Unlike transposon recombination, V(D)J recombination requires a second apparently unrelated protein denoted RAG-2 protein [5]. Without RAG-2 protein, RAG-1 is not functional as a recombinase *in vivo*. Genes encoding RAG-1 and RAG-2 proteins are closely linked in inverted orientation in the genome of vertebrates that have acquired immune systems and the genome of the sea urchin that does not have an acquired immune system [7]. It would support the “RAG-transposase” model if a transposon containing a transib like transposase also contained a RAG-2 like protein. However, no such transposon has been found. This might be termed the RAG-2 problem.

**Figure 4:**
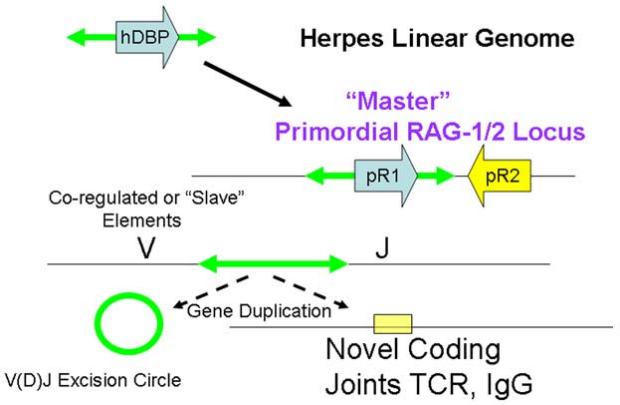
The Co-regulatory model including initial insertion of a primordial herpes virus recombinase (proto-RAG-1 denoted pR1) adjacent to a pre-existing RAG-2 like protein (denoted pR2) is shown. As shown, insertion of a herpes virus episome or linear genome adjacent to a RAG-2 like gene would provide a master co-regulated RAG-2/RAG-2 locus acting subsequently through co-evolving slave RSS sites in immunoglobulin or T-cell receptor genes. Co-evolving slave RSS could arise either from additional herpes or transposon insertions and gene duplication events or from co-evolution of endogenous sequences with some similarity to transposon or herpes virus termini in other genes such as those encoding B- and T-lymphocyte receptors (Figure 1). In contrast to the “RAG transposon” model, the co-regulatory model does not require the existence of a composite RAG-1/RAG-2 transposase or transposon and can also account for the experimental structure of the current RAG-1/RAG-2-like genes in the sea urchin and other deuterostomes that do not undergo V(D)J recombination.

A co-regulatory model of the origins of V(D)J recombination (Figure 4) would require only a herpes-like element insertion generating a master regulatory site with a primordial RAG-1-like recombinase (denoted pR1) adjacent to a pre-existing RAG-2-like protein (denoted pR2). Regulated expression of this primordial RAG-1/RAG-2 complex in lymphocytes that could then co-evolve gradually with subsequent “slave elements” arising independently in T-cell receptor and immunoglobulin genes. In this “co-regulatory model” the possible homology between herpes recombination and V(D)J recombination are of critical importance because, unlike simple DNA transposable elements such as the Tc and transib elements which are regulated by the sequences flanking the element, herpes viruses insert into genomes with cis-acting regulatory sequences.

Notably, DNA binding proteins of the EBV replication complex and related proteins of other herpes viruses are highly antigenic proteins [39], [40] composing the so called “early antigen.” Expression of a herpes-like protein or proteins with regulated expression in the lymphocytes of an organism could immediately provide a potential selective advantage to the individual through stimulation of pathogen specific pattern receptors of the innate immune system such as toll receptors present in the sea urchin and similar invertebrates. Primordial RAG-2 protein, co-expressed with RAG-1 and co-selected initially as a repressor of recombination could then gradually co-evolve with RAG-1 in the somatic immune system with distinct sequences in the immunoglobulin and T-cell receptor genes.

In contrast, if a transib-like transposable element did insert at multiple sites in the germ line sea urchin, for example at separate sites in primordial T cell receptor gene and an immunoglobulin precursor gene (the so-called RAG transposon model), this must have been followed by subsequent loss of all traces of these elements at multiple sites except at their termini, while an another complete copy of the element remained elsewhere in the genome. Also in contrast, there would be no apparent selective advantage of a transposase expressed in lymphocytes prior to the origins of V(D)J recombination, and in fact such a recombination prone site might be selected against due random chromosomal breakage and recombination at endogenous transib like sequences and sequences in somatic herpes viruses [41]–[43].

### Evidence in Support of the Herpes Co-Regulation Model; Cis-acting regulatory sequences adjacent to the herpes DBP BALF-2 are sufficient to confer response to the V(D)J recombination activating signal ligation of surface IgG

Most critically, herpes virus genomes contain their own regulatory sequences, which enable them to sense the environment of the host cells in which they reside and interact with complex regulatory networks in host cells [44]. Although DNA transposons also interact with cellular regulatory networks, DNA transposons such as the transib elements do not encode these networks themselves, but rather are regulated by the genes into which they insert [45], [46]. Although one class of DNA transposon has been described with multiple genes and internal regulatory sequences, this element is more similar to a DNA virus (such as a herpes virus) than a transib element [47].

**Figure 5:**
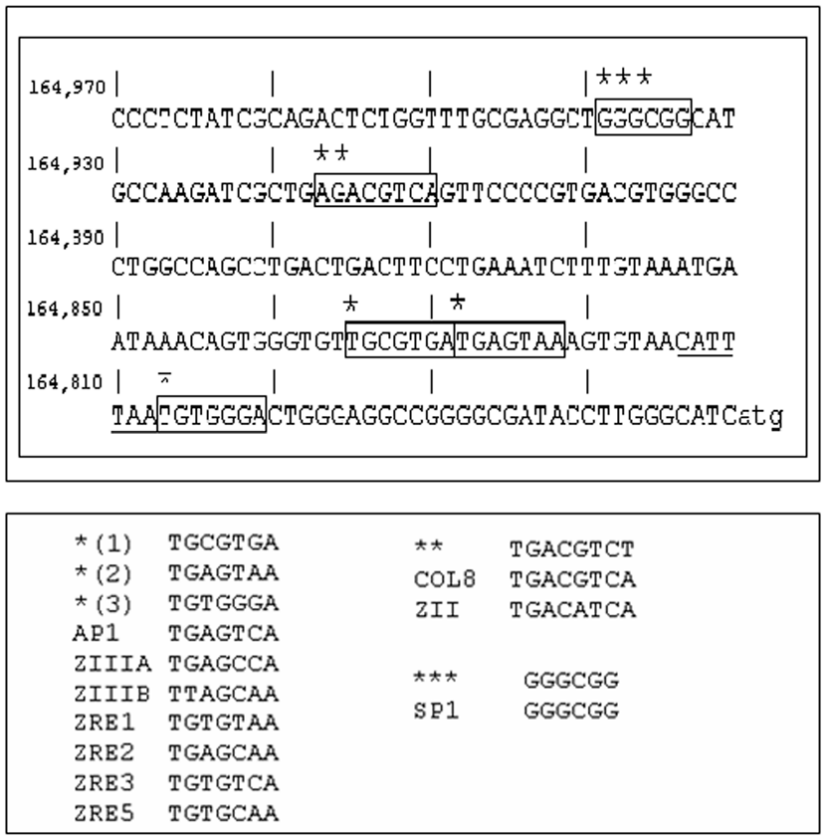
Shared somatic regulation between the EBV DBP BALF-2 protein gene and RAG. As shown, a 200 NT 5′ region immediately adjacent to EBV BALF-2 ORF AUG start codon contains putative regulatory sequences for BZLF-1/AP-1 (denoted with a single asterisk), CREB (denoted with a double asterisk**), and SP1 (denoted with a triple asterisk***). These putative regulatory sequences are enclosed in boxes in the figure and include sequences recognized by the EBV encoded BZLF-1 regulatory protein (also termed ZEBRA protein). BZLF-1 sites also are also functional as sites for the endogenous regulatory factor AP-1 as discussed in the text. BZLF-1 regulated sites from other EBV genes ZIIIA, B, and ZRE1,2,3,5 are shown in comparison to a consensus AP-1 site in the lower portion of the figure. Similarly, in the lower portion of the figure the putative binding site for CREB is shown in the BALF-2 minimal promoter in comparison to Col8, a cAMP response element shown to bind CREB1 cAMP site binding protein with high affinity and ZII, a site in the BZLF-1 promoter shown experimentally to respond to cAMP. Also in the lower portion of the figure, a site in the BALF-2 minimal promoter matching the Sp1 transcription factor consensus is shown, similar but not identical to Sp1 binding sites confirmed to exist in the EBV terminal repeats (Figure 2).

Cis-acting regulatory sequences immediately adjacent to the herpes DBP such as the BALF-2 protein of EBV, the only DBP for which experimental data is also available [48] have sequences resembling response elements for cellular factors such as AP-1 and also elements resembling other sites recognized by host transcription factors such as cAMP (cyclic AMP) and SP1 and AP-1 (Figure 5). These same transcription factor families also regulate expression of the RAG proteins [49]. Sites for viral activation factor BZLF-1 protein binding resemble sites for the AP-1 transcription factor [34], [35], [50]. Cis-acting regulatory sequences present within approximately 2 kb of the BALF-2 ORF adjacent to the herpes DBP BALF-2 from EBV previously have been demonstrated to respond to the viral transcription factor BZLF-1 [48]. As shown in Figure 5 of these BZLF-1 responsive sequences can further be localized within a 200 base region immediately adjacent to the BALF-2 ORF [48].

These previous experiments have identified the BALF-2 transcription start site (nt 164,782 of standard reference EBV B-958 strain EBV genome) and characterized BZLF-1 sites in the region −134 to −64 contained in the 200 bp shown in Figure 5 by deletion analysis of the promoter and gel shift [48]. Functional binding and transcription activation of this region by viral BZLF-1 protein binding (functionally equivalent to host AP-1 binding) was confirmed in these studies. An additional functional transcription site upstream of the 200 bp region shown was identified for Rta, an EBV encoded viral transcription factor distinct from AP-1/BZLF-1 protein and characterized just 5′ of the 200 bp region (−287 to −254). These previous studies were confirmed in this work by demonstrating that the BALF-2 minimal promoter region shown in Figure 5 is significantly activated both by viral and cellular AP-1 transcription factors (unpublished data).

In addition to AP-1 transcription factors, RAG protein expression is also regulated by cAMP expression in lymphocytes [49]. Sequences resembling a cAMP response element are present in EBV BALF-2 minimal promoter region (Figure 5). This region of the BALF-2 promoter was studied by gel shift analysis using CREB1 protein expressed and purified from bacterial cells which formed a nucleoprotein complex with the BALF-2 CRE-like sequence shown. A specific nucleoprotein complex was detected in *in vivo* gel shift experiments in Akata cells and other EBV positive lymphoblastoid cell lines with the BALF-2 CRE like element shown ( CREB binding unpublished data).

RAG protein expression is also regulated by the physiologic signals generated by ligation of surface IgG, for example to initiate receptor editing of self-reactive immunoglobulin molecules [51], [52] and similar process may edit the T-cell receptor [53]–[57]. Infection of of both B and T-lymphocytes has also been shown to result in a robust co-stimulation of RAG expression *in vivo*, suggesting that regulatory networks are shared between EBV and RAG expression [17], [19], [31]. Ligation of surface IgG in Akata cells is sufficient to activate sequences from the BAFL-2 promoter shown in Figure 5 and also to cause expression of the BALF-2 protein in EBV infected lymphoblastoid cells as detected by Western Blotting (unpublished data). A correlation between BALF-2 expression and viral recombination was also supported by the presence of intra-nuclear BALF-2 protein in EBV positive lymphocyte cell lines permissive for viral replication, consistent with localization of herpes DBP to the nucleus during viral replication [58]. Thus a very minimal cis-active promoter residing in 200 nucleotides 5′ of the BALF-2 protein coding sequence is sufficient to coordinate endogenous cellular transcription factors including AP-1, CREB, and SP1 in response to ligation of surface IgG in human B lymphocytes resulting in co-ordination of expression of BALF-2 and RAG protein expression.

### The RAG-1 amino terminus problem

**Figure 6:**
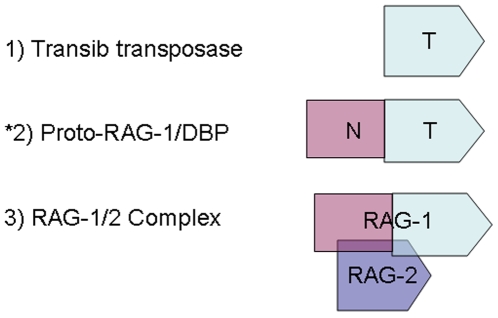
The hypothesis that a herpes DBP-like protein and RAG-1 protein have a modular architecture with structural and functional homology of functions is presented. Primordial RAG-1 protein (denoted pR1) has a carboxyl region structurally similar to a transib transposase (denoted T for Transib-like region structure #1), but extra amino terminus protein sequences that may be derived from another protein family (denoted N). Herpes DBP are magnesium dependent recombinases are also modular proteins with an amino terminal regulatory region (denoted N), and a carboxyl terminus that binds to DNA. The RAG-1 protein currently requires a physical association with the RAG-2 protein for recombinase activity *in vivo*, but may have initially exhibited recombinase properties without RAG-2 analogous to the DBP. As discussed in the text, primordial RAG-2 protein (denoted pR2) may initially have blocked the recombinase functions of pR1 but exposed immunologic determinants essential to herpes virus immunity since the DBP are a major herpes virus antigen. Both Herpes DBP and RAG-1 also require an association with host cell factors such as DNApk and ku shared with the RAG proteins for viral recombinase activity *in vivo* as discussed in more detail in the text.

Transposons of the Transib family have been proposed to be the core of a “RAG transposon” encoding the core recombinase functions [6] of RAG-1. However, RAG-1 protein also has a modular structure with amino terminal sequences derived from an apparently unrelated protein family (Figure 6). While it is plausible that a transib element could have transposed to its current site adjacent to RAG-2 as part of a transposition event mediated by a transib-like transposase, there are current no known transposons with a RAG-1 like transposase including N-terminal sequences as a functioning transposon. How then would a transib-like element simultaneously acquire amino terminal sequences from a host gene and get to a site in the sea urchin genome adjacent to the RAG-2 gene ? This might be termed the RAG-1 amino terminus problem.

As shown in Figure 6, the herpes DBP and the RAG-1 protein both have a modular structure with an N-terminal regulatory domain and a C-terminal DNA binding domain. In this work it is proposed that both herpes DBP and the RAG proteins have a similar modular structure because both protein descended from a common ancestral herpes-like recombinase proto RAG-1 ( pR1, Figure 6). A putative herpes virus recombinase pR1 homologous to RAG-1 with additional N-terminal amino acid sequences in both proteins [17] would also provide amino terminal protein sequences present in RAG-1, and bind to a primordial RAG-2 protein (pR2) co-expressed in somatic cells prior to the origins of the acquired immune system in the sea urchin. As proposed for pR1, the function of amino terminal sequences in the herpes ICP-8 protein are not directly related to DNA binding properties of the protein but rather seem to associate with cellular factors and regulate other viral genes [58]–[62].

The function of amino terminal sequences in RAG-1 protein remains unresolved since in fact they can be deleted to yield a core transposase capable of in vitro V(D)J recombination [6]. It is plausible that the amino terminal regions of RAG-1 and pR1 (Figure 6) could also bind to other factors distinct from the recombination properties of the protein. Factors such as ku and DNA pk involved in non-homologous repair of RAG-1 generated DNA breakage prior to DNA replication [63], [64] are also associated with the herpes DBP ICP-8 [32].

Both herpes virus replication and V(D)J recombination (but not transib element transposition) occur synchronously during the G0/G1 phase of the cell cycle coordinately with V(D)J recombination, and co-coordinating interactions with cell cycle regulatory proteins [32] might also be a function of the amino terminus of herpes DBP shared with amino terminal regions of RAG-1. Similar amino terminal regions shared between RAG-1 and the herpes DBP thus could thus prevent interactions between DNA synthesis of the host and transib transposition during the S phase of the cell cycle, preventing chromosomal fragmentation at dispersed transib-like sequences (Figure 1) from occurring if V(D)J recombination occurred during the S phase.

### Additional structural and sequences similarity between herpes DBP and RAG-1

No primary sequence similarity is evident between DBP and RAG-1 using conventional algorithms such as BLAST or publicly available structural software (unpublished observations). However, it might be expected that if the primary selective advantage of a primordial herpes virus genome insertion into the germ line genome was to provide an augmented immunologic response to subsequent herpes infections in descendant organisms then in turn herpes viruses would rapidly alter their primary sequences and immunologic determinants obscuring primary sequence similarity as a consequence of the “arms race.” Such an immunologic “arms race” would not alter secondary and tertiary functional relationships between the proteins. For comparative purposes, it would be helpful if the complete crystal structure of both RAG and DBP proteins were solved so that, for example the location of the respective DNA binding sites and magnesium binding sites could be compared. Unfortunately the RAG-1 structure is not solved, although some structural features have been inferred theoretical structures derived through computational modeling and through comparison with invertebrate transposases [65], [66].

**Figure 7:**
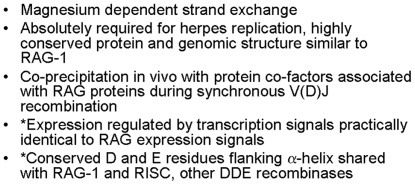
Summary of functional correlates between RAG-1 and herpes DBP (asterix indicates observations novel to this work, other observations presented previously). These functional correlates are consistent with a homologous descent of both proteins from a common precursor recombinase although analogous convergence of functional properties cannot be excluded.

Both RAG-1 and herpes DBP are similar in size and biochemical properties such as non-specific DNA binding due to numerous highly acidic amino acid residues, as well as magnesium dependent DNA binding and strand exchange reactions in vitro [17], [23]. A zinc binding finger is present in a similar region of RAG-1 and the DBP, however zinc sites are present in many DNA binding proteins [17]. A summary of relevant functional similarities between herpes virus DBP and the RAG-1 protein is provided in Figure 7. In all cases in which functional similarities have been looked for between the DBP and RAG proteins they have been identified, despite the absence of primary sequence similarities between these protein families.

In addition, in this work it is shown that some residual primary sequence similarities may still be evident between the DBP and RAG-1 protein , particularly in functional domains of the two proteins that have a high “information content” in contrast to the less conserved non-functional or spacer regions of the proteins. The author has previously noted [67] that improved search algorithms based upon conserved function and “information content” are needed to assess similarity of proteins in cases in which strong evolutionary selection is suspected. As noted previously by the author in the case of the homologous related p53 tumor suppressor and NF-κB transcription factor protein families, important functionally conserved regions of homologous protein families are not evident unless “information content” including weighting of structural and functional regions of the proteins are assessed in alignment algorithms [67].

**Figure 8:**
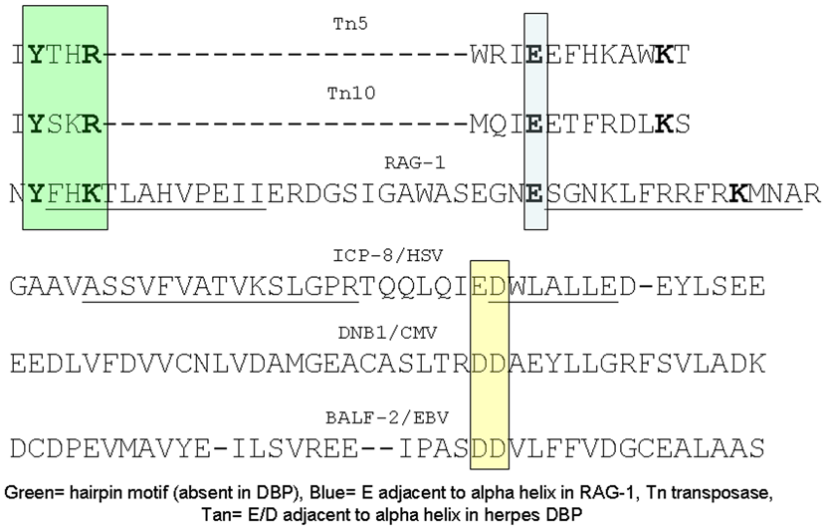
Conserved Functional DDE residues between transposases, RAG proteins and herpes DBP. Despite primary “low information content” amino acid sequence divergence of intervening sequences, RAG-1 proteins encode a “high information content” absolutely conserved E residue adjacent to a conserved alpha helix in the extreme carboxyl terminus of the protein shared with prokaryotic transposons (Tn5 and Tn10). This functionally conserved residue is required for RAG-1 magnesium ion binding and protein function. Similarly, despite primary amino acid sequence divergence of intervening regions all herpes DBP encode a conserved D/E residue adjacent to a conserved alpha helix in the DNA-binding carboxyl terminus of the protein. These high information content similarities are consistent with and support descent of both proteins from a common precursor recombinase.

Recently a partial crystal structure of a herpes DBP has been solved, although lacking the carboxyl terminus of the protein and some of the magnesium ion binding properties of the protein determined [21], [22]. As shown in this work (Figure 8), both the theoretical structures of RAG proteins and the partially solved structure of the herpes DBP ICP-8 share highly conserved D and E residues at the borders of alpha helical regions in the carboxyl terminus of the proteins. For example, at the border of alpha helix 29 and 30 of the DBP proteins in the carboxyl region of the protein two sequential conserved D or E residues are found in all DBP, while a conserved D in the corresponding carboxyl region of RAG-1 parties the terminal conserved E of the experimentally conserved DDE triad binding a magnesium ion (Figure 8).

The author proposes that these conserved D and E residues, as well as other conserved structural features have a very high “information content,” and thus are relatively more important in supporting protein homology, while other sequences less conserved even between different viral DBP function as relative sequence independent spacer elements aligning the structural regions and thus have a low sequence “information content.” Because of the high degree of primary sequence divergence of alpha, beta, and gamma herpesviridae no other residues of all three DBP are conserved in this or most other regions of the respective DBP proteins (Figure 8).

An indirect suggestion that these regions of the DBP are involved in magnesium ion binding is that disruption of the carboxyl terminus of the DBP seems to eliminate magnesium ion binding since magnesium ion is not noted in the partially solved ICP-8 structure with carboxyl deletions [22]. These observations are consistent with an immunologic arms race between a primordial herpes virus and an inserted copy of the virus in which the virus would exhibit conservation of “high information content” structural domains with rapid divergence of primary sequence similarity. As structural information about the RAG and DBP becomes available in the future, an important prediction of the co-regulatory hypothesis proposed in this work is that further “high information content” structural and functional correlations of the two families of proteins will become evident, although these similarities are not evident through conventional comparisons of primary amino acid sequences.

### Structural Similarity Between Herpes DBP ICP-8 and another vertebrate protein, Argonaute containing a DDE/RNAse H catalytic site

Although the structure of RAG-1 is not determined and thus cannot be directly compared to the partial ICP-8 structure, a complete structure has been determined for the Argonaute protein, a component of the double stranded RNA nuclease RISC (RNA Induced Silencing Complex) [25]. RISC proteins such as Argonaute bind to single and double stranded RNA and direct site-specific cleavage of the bound RNA. The RISC protein component termed Argonaute, like RAG-1, is a DDE-family recombinase in which magnesium ions are bound to conserved acidic residues [24], [68]. In the complete RISC structure a DNA binding groove aligns a double stranded nucleic acid (RNA) with a DDE bound magnesium ion so that the magnesium ion contacts and cuts the nucleic acid at a defined site (RISC data not shown, available in references cited by Leemor Joshua-Tor et al.).

**Figure 9:**
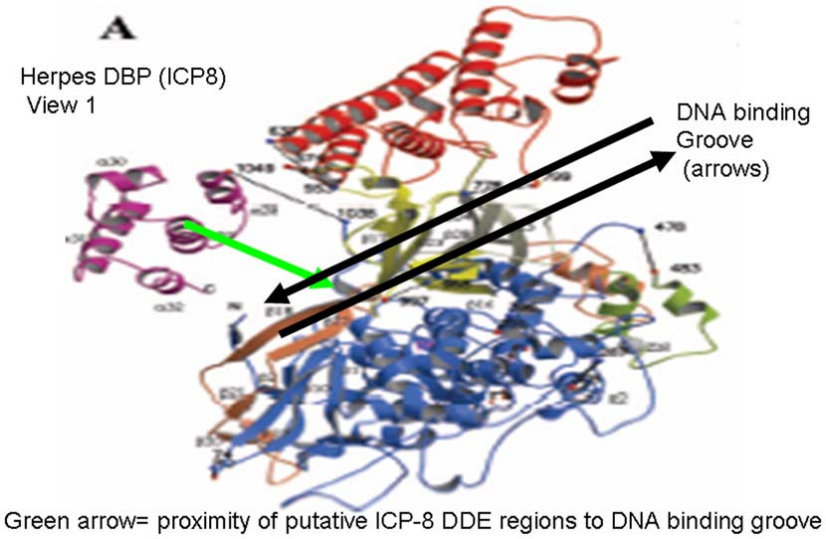
Putative magnesium ion binding regions of the DBP can be localized adjacent to the DNA binding groove of the ICP-8 protein structure. The partial crystal structure of herpes simplex DBP ICP-8 is shown with experimentally determined DNA binding groove shown, while experimentally determined structures of RAG proteins and other herpes DBP are not solved currently. A black double arrow illustrates the experimentally determined DNA binding groove of ICP-8, while a green arrow indicates the hypothetical position of a bound magnesium ion in ICP-8 as localized by conserved blocks of D and E residues shared with RAG-1 in regions of ICP-8 (Figure 8). This alignment shows that the predicted Mg binding site geometry of ICP-8 is in proximity to the bound DNA as in other structurally characterized DDE enzymes such as RISC. These structural similarities are consistent with and support descent of DBP and RISC proteins from a common precursor DDE recombinase.

Some functional properties are clearly co-localized in the ICP-8 partial structure and the complete RISC structure, and the architecture of the proteins is similar although no primary sequence similarity is evident between RISC and either the herpes DBP or the RAG-1 protein. . Most notably the groove formed in RISC that binds a double stranded RNA is quite similar in orientation and structural elements to the groove identified as the DNA binding region of ICP-8 in the ICP-8 partial structure. Conserved D and E residues in ICP-8, other herpes DBP and RAG proteins (defined in Figure 8) are in proximity and thus capable of contacting a bound double stranded nucleic acid based upon their positions in the partial ICP-8 structure (Figure 9). In this alignment the hypothetical positions of a magnesium binding site and site of double stranded DNA binding in the ICP-8 protein are shown, potentially orienting a bound magnesium ion towards the bound DNA strand to permit, for example, magnesium dependent strand exchange typical of DBP proteins such as ICP-8. Most importantly, the predictions of this model of the ICP-8 protein are empirically testable because mutations in the putative DDE site of ICP-8 and related herpes DBP (Figure 8, 9) should both eliminate magnesium binding of the DBP protein and also inactivate function of the DBP protein in DNA strand exchange and herpes virus replication without altering other functional properties of the DBP protein.

## Discussion

**Figure 10:**
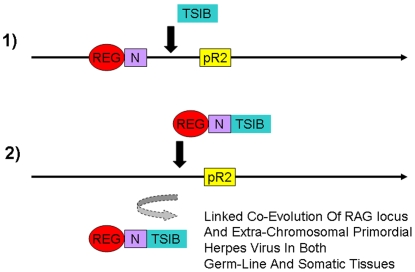
Possible scenarios for origins of V(D)J recombination in the absence of a “RAG Transposon.” The co-regulatory hypothesis presented in this work cannot exclude the possibility that a transib transposon-like element inserted directly into the current RAG locus adjacent to a primordial RAG-2 gene and a conveniently located independent N terminal-like protein with sequences somatically regulated in immune cells already present at the site as shown in the top scenario (Scenario 1). However, this scenario would require several independent coincidences of adjacent N protein and regulatory sequences adjacent to proto RAG2 not found experimentally. Scenario 1 also provides no explanation for the continued presence of the RAG-1/RAG-2-like locus in the modern sea urchin genome in the absence of any known function or slective advantage. In a more probable scenario shown in the bottom panel a herpes-like episome already containing N terminal protein sequences and cis-linked somatic regulatory sequences inserted adjacent to a primordial RAG-2 protein to generate the current RAG site (Scenario 2). After the initial generation of the RAG site in either scenario, the initial selective benefit of the RAG locus may have been to provide immunity to subsequent herpes virus infection rather than V(D)J recombination for an undetermined interval of time during which herpes and RAG protein primary sequences diverged, and this locus may still provide some partial immunity until the present time to conserved “high-information content” regions of the herpes recombinase that cannot diverge due to functional constraints . After the primordial herpes virus lineage had diverged sufficiently in primary sequence to permit re-infection of the primordial deuterostome host with herpes-like pathogens, resumption of the herpes-host arms race would continue until the present.

Does the RAG transposon exist, or is it like the Unicorn, a literary icon for the faithful [14]? This is not an unimportant question, because the “RAG transposon model” is currently the only published model of the origins of the acquired immune system, and yet the RAG transposon has not been located despite an intensive search as reviewed in this manuscript. An alternative and radically different model is suggested in this work that can “save the phenomena” with a minimum of ad hoc postulates. This model has experimentally testable consequences, since, unlike the RAG transposon, herpes viruses exist in the biological world. In the “co-regulatory model proposed in this work, stated simply, the RAG transposon does not exist. Instead a mobile sequence similar to a modern day herpes virus encoding a recombinase core similar to a transib transposon but also with additional amino terminal somatic regulatory sequences (termed pRAG-1) inserted adjacent to primordial RAG-2 in the germ line of species lacking V(D)J recombination or an acquired immune system (Figure 10).

This event introduced cis-acting genetic regulatory sequences capable of co-regulating expression of hypothetical proto-RAG-1 and proto-RAG-2 proteins (Figure 5). Additional co-regulation of the pRAG-1 protein in somatic tissues was provided by amino terminal regulatory sequences similar to those shared between the modern herpes DBP and RAG proteins (Figure 6). Co-expressed proteins in somatic immune cells were subsequently selected positively through their stimulation of innate immunity to herpes infection through interactions with pattern recognition toll-like receptors facilitating co-evolution of the two proteins. The inserted viral recombinase may have been initially blocked in somatic cells through association with a co-expressed RAG-2-like protein (Figure 7) preventing the teratogenic or mutagenic properties of the viral recombinase protein, but not altering immunologic determinates and thus providing a selective advantage to the ancestral deuterostome. Subsequently, further co-evolution of RAG-1 and RAG-2 provided the partially unblocked recombinase functions active against endogenous slave elements or V(D)J like sequences in endogenous genes required for somatic generation of the immunoglobulin and T-cell repertoires in vertebrates, but not in other descendants of the ancestral deuterostome such as the modern sea urchin.

Currently, the sea urchin genome encodes more than 20 toll-like receptors in contrast to approximately 10 in the human genome, as well as numerous other pattern recognition and signaling molecules in the innate immune pathway, suggesting that innate or pattern recognition elements played an important role in the immunity of ancestral deuterostomes. Although a specific receptor required innate immunity to herpes viruses in the co-regulatory model has not been identified in this work, identification of such a molecule would support the co-regulatory model proposed in this work.

As reviewed in this work, cis-acting regulatory sequences adjacent to a herpes virus recombinase DBP encoded by EBV are able to regulate adjacent genes with in response to cellular factors such as AP-1 and cAMP, and also in response to much more complex physiologic stimuli such as ligation of surface IgG. Transcription factors are ubiquitous, functional in not only the hematopoetic lineage but many other cell types, thus the conservation of theses sequences in both the RAG and DBP promoters does not in and of itself confirm a homology of the two pathways, but is consistent with such a model. Conservation of physiologic signaling pathways such as co-expression of both RAG and DBP following ligation of surface IgG are much more complex than simply the presence of these common regulatory elements and more strongly support a homologous rather than analogous relationship between the regulatory pathways.

Insertion of a RAG-1 like DDE recombinase adjacent to a primordial RAG-2 like regulatory protein would permit the co-expression and co-evolution of primordial RAG-1 and RAG-2 in somatic tissues of the lymphocyte lineage through a molecular “arms race” between herpes viruses and the ancestral immune deuterostome immune system . The modern herpes virus families would then diverge in primary sequence to evade the ancestral deuterostome innate immune system as noted , and develop other evasive mechanism such as encoding immuno-suppressive cytokines (such as an EBV encoded IL-10 homologue) while maintaining conserved structural and functional sites.. In the herpes viruses, the functional DDE site (Figure 8, 9) would continue to serve as a viral recombinase. In the ancestral deuterostome genome the putative DBP related RAG-1 DDE site, at first blocked by the proto-RAG-2 protein co-expressed in immune somatic cells would over time evolve to form the nucleus of a somatic recombinase generating a variable repertoire against pathogens such as herpes viruses.

Possibly, a DDE site in the modern sea urchin RAG-1-like protein, in the absence of the sea urchin RAG-2 like protein, may still retain some recombinogenic functions on herpes-like termini identified in this work , transib element termini, or even modern V(D)J sequences. Conversely, all recombinogenic functions of the modern sea urchin RAG-1 like molecule may have been lost over time, with the RAG-like locus maintained over time only as an antigenic stimulus or with some other, as yet unknown function in for example cell cycle regulation. With respect to the herpesviridae, further divergence of modern day herpes viruses specialized for episomal replication and persistence in lymphocytes and other somatic tissues would then result in this model from capture of additional host genes, including additional cellular regulatory and structural proteins also termed the “Ping Pong” model of herpes genome structure.

Gamma herpes viruses such as EBV continue to replicate in the cells of the immune system, while other herpes viruses in the alpha and beta herpes families (Figure 8) have diverged into specific somatic niches over time are not capable of replication or productive gene expression in hematopoetic cells. Since EBV has retained the hematopoetic regulatory sequences co-regulated with the RAG genes as reviewed in this work, it is proposed that EBV sequences present in the BALF-2 promoter currently most closely resemble the primordial regulatory sequences inserted adjacent to proto-RAG-2. Thus further comparative analysis of EBV and RAG expression may provide evidence in support of the co-regulatory model presented in this work, already suggested by the oncogenic consequences of EBV infection through co-activation of pathogenic V(D)J recombination.

In contrast to the hypothetical and possibly fictional “RAG transposon,” a number of readily testable hypotheses regarding the structural similarities between the RAG-1 protein and herpes DBP (in the absence of primary sequence similarity) are also inferred from these observations. Some of these predictions include 1) Mutation of conserved D and E residues in the carboxyl region of the herpes DBP such as ICP-8 (Figures 8, 9) should eliminate magnesium bind to the protein and hence eliminate herpes recombination and replication equivalent to that observed with complete deletion of the ICP-8 protein analogous to the effects of elimination of these residues in the RAG-1 protein. 2) Localization of the functional magnesium binding site of the DBP should co-respond with conserved D and E residues in the DBP as well as corresponding regions of the RAG-1 protein. 3) Other functional properties of the DBP such as association with Ku, DNA pK should occur through common regions of the DBP and RAG-1protein 4) Complete crystal structures of the DBP and RAG-1 when available, or theoretical equivalents should have functional conservation domains as suggested in this work. 5) The amino-terminal regions of RAG-1 and DBP proteins, proposed in this work to interact with cellular factors regulating the cell cycle based on studies of the herpes DBP, may be at least in part functionally conserved and hence interchanged , for example between RAG-1 and BALF-2, without affecting protein function if the role of these amino terminal sequences is to confer cell cycle regulation and other regulatory properties in somatic cells 6) Evidence of an association between current vertebrate RAG-2 proteins or the RAG-2 like protein encoded in the sea urchin genome and the current viral DBP such as BALF-2 may still be evident and exhibit the recombination blocking role predicted in the co-regulatory model. 7) Homologous interacting and thus interchangeable protein sequences may also be shared between the carboxyl terminus of RAG-1 and the amino terminus of RAG-2 as well as the herpes DBP as a consequence of the original insertion event [69]

Obviously, it will not be possible to revisit the origins of the acquired immune system except through empirically testable hypotheses. Since the “RAG-transposon” has not been located despite an extensive search, it is time to evaluate the origins of the acquired immune system critically and experimentally rather than as dogma. The so-called “co-regulatory hypothesis” provided in this work although incomplete and preliminary in nature provides experimentally verifiable connections between the herpes viruses and the RAG proteins. In summary the co-regulatory hypothesis : 1) suggests a reason for the observed structure of the RAG-1 and RAG-2 locus in the sea urchin, apparently predating V(D)J recombination; 2) co-relates the known functional properties of the current acquired immune system and the herpes viruses as well as numerous regulatory features shared between these elements; 3) provides an explanation for shared regulatory network and mutagenic potential of both herpes virus replication and V(D)J recombination and the complex and possibly intersecting roles of these pathways as co-factors in human malignancy through V(D)J recombination pathogenesis. 4) suggests novel strategies for altering the replication of modern day herpes viruses since these agents apparently recombine through a process analogous to the formation of immunoglobulin and T cell receptor excision episomes.

## Materials and Methods

This manuscript is a theoretical treatment of previously published and unpublished observations to be published elsewhere.

## References

[1] Tonegawa S (1983). Somatic generation of antibody diversity.. Nature.

[2] Dreyfus DH (1992). Evidence suggesting an evolutionary relationship between transposable elements and immune system recombination sequences.. Mol Immunol.

[3] Spanopoulou E, Zaitseva F, Wang FH, Santagata S, Baltimore D (1996). The homeodomain region of Rag-1 reveals the parallel mechanisms of bacterial and V(D)J recombination.. Cell.

[4] Jones JM, Gellert M (2004). The taming of a transposon: V(D)J recombination and the immune system.. Immunol Rev.

[5] Schatz DG (2004). Antigen receptor genes and the evolution of a recombinase.. Semin Immunol.

[6] Kapitonov VV, Jurka J (2005). RAG1 core and V(D)J recombination signal sequences were derived from Transib transposons.. PLoS Biol.

[7] Fugmann SD, Messier C, Novack LA, Cameron RA, Rast JP (2006). An ancient evolutionary origin of the Rag1/2 gene locus.. Proc Natl Acad Sci U S A.

[8] van Gent DC, Mizuuchi K, Gellert M (1996). Similarities between initiation of V(D)J recombination and retroviral integration.. Science.

[9] Melek M, Jones JM, O'Dea MH, Pais G, Burke TR (2002). Effect of HIV integrase inhibitors on the RAG1/2 recombinase.. Proc Natl Acad Sci U S A.

[10] Hiom K, Melek M, Gellert M (1998). DNA transposition by the RAG1 and RAG2 proteins: a possible source of oncogenic translocations.. Cell.

[11] Agrawal A, Eastman QM, Schatz DG (1998). Transposition mediated by RAG1 and RAG2 and its implications for the evolution of the immune system.. Nature.

[12] Shih IH, Melek M, Jayaratne ND, Gellert M (2002). Inverse transposition by the RAG1 and RAG2 proteins: role reversal of donor and target DNA.. Embo J.

[13] Chatterji M, Tsai CL, Schatz DG (2004). New concepts in the regulation of an ancient reaction: transposition by RAG1/RAG2.. Immunol Rev.

[14] Brandt VL, Roth DB (2008). G.O.D.'s Holy Grail: discovery of the RAG proteins.. J Immunol.

[15] Tsai CL, Schatz DG (2003). Regulation of RAG1/RAG2-mediated transposition by GTP and the C-terminal region of RAG2.. Embo J.

[16] Swanson PC, Volkmer D, Wang L (2004). Full-length RAG-2, and not full-length RAG-1, specifically suppresses RAG-mediated transposition but not hybrid joint formation or disintegration.. J Biol Chem.

[17] Dreyfus DH, Kelleher CA, Jones JF, Gelfand EW (1996). Epstein-Barr virus infection of T cells: implications for altered T-lymphocyte activation, repertoire development and autoimmunity.. Immunol Rev.

[18] Kuhn-Hallek I, Sage DR, Stein L, Groelle H, Fingeroth JD (1995). Expression of recombination activating genes (RAG-1 and RAG-2) in Epstein-Barr virus-bearing B cells.. Blood.

[19] Srinivas SK, Sixbey JW (1995). Epstein-Barr virus induction of recombinase-activating genes RAG1 and RAG2.. J Virol.

[20] Wagner HJ, Scott RS, Buchwald D, Sixbey JW (2004). Peripheral blood lymphocytes express recombination-activating genes 1 and 2 during Epstein-Barr virus-induced infectious mononucleosis.. J Infect Dis.

[21] Makhov AM, Taylor DW, Griffith JD (2004). Two-dimensional crystallization of herpes simplex virus type 1 single-stranded DNA-binding protein, ICP8, on a lipid monolayer.. Biochim Biophys Acta.

[22] Mapelli M, Panjikar S, Tucker PA (2005). The crystal structure of the herpes simplex virus 1 ssDNA-binding protein suggests the structural basis for flexible, cooperative single-stranded DNA binding.. J Biol Chem.

[23] Dreyfus DH (2006). The DDE recombinases: diverse roles in acquired and innate immunity.. Ann Allergy Asthma Immunol.

[24] Song JJ, Smith SK, Hannon GJ, Joshua-Tor L (2004). Crystal structure of Argonaute and its implications for RISC slicer activity.. Science.

[25] Liu J, Carmell MA, Rivas FV, Marsden CG, Thomson JM (2004). Argonaute2 is the catalytic engine of mammalian RNAi.. Science.

[26] Dyda F, Hickman AB, Jenkins TM, Engelman A, Craigie R (1994). Crystal structure of the catalytic domain of HIV-1 integrase: similarity to other polynucleotidyl transferases.. Science.

[27] Chowdhury SI, Buhk HJ, Ludwig H, Hammerschmidt W (1990). Genomic termini of equine herpesvirus 1.. J Virol.

[28] Hammerschmidt W, Sugden B (1990). DNA replication of herpesviruses during the lytic phase of their life-cycles.. Mol Biol Med.

[29] Zimmermann J, Hammerschmidt W (1995). Structure and role of the terminal repeats of Epstein-Barr virus in processing and packaging of virion DNA.. J Virol.

[30] Kolman JL, Kolman CJ, Miller G (1992). Marked variation in the size of genomic plasmids among members of a family of related Epstein-Barr viruses.. Proc Natl Acad Sci U S A.

[31] Srinivas SK, Sample JT, Sixbey JW (1998). Spontaneous loss of viral episomes accompanying Epstein-Barr virus reactivation in a Burkitt's lymphoma cell line.. J Infect Dis.

[32] Taylor TJ, Knipe DM (2004). Proteomics of herpes simplex virus replication compartments: association of cellular DNA replication, repair, recombination, and chromatin remodeling proteins with ICP8.. J Virol.

[33] Delecluse HJ, Hammerschmidt W (2000). The genetic approach to the Epstein-Barr virus: from basic virology to gene therapy.. Mol Pathol.

[34] Carey M, Kolman J, Katz DA, Gradoville L, Barberis L (1992). Transcriptional synergy by the Epstein-Barr virus transactivator ZEBRA.. J Virol.

[35] Francis AL, Gradoville L, Miller G (1997). Alteration of a single serine in the basic domain of the Epstein-Barr virus ZEBRA protein separates its functions of transcriptional activation and disruption of latency.. J Virol.

[36] Moody CA, Scott RS, Su T, Sixbey JW (2003). Length of Epstein-Barr virus termini as a determinant of epithelial cell clonal emergence.. J Virol.

[37] Spain TA, Sun R, Miller G (1997). The locus of Epstein-Barr virus terminal repeat processing is bound with enhanced affinity by Sp1 and Sp3.. Virology.

[38] Sun R, Spain TA, Lin SF, Miller G (1997). Sp1 binds to the precise locus of end processing within the terminal repeats of Epstein-Barr virus DNA.. J Virol.

[39] Paramita DK, Fachiroh J, Artama WT, van Benthem E, Haryana SM (2007). Native early antigen of Epstein-Barr virus, a promising antigen for diagnosis of nasopharyngeal carcinoma.. J Med Virol.

[40] Dardari R, Hinderer W, Lang D, Benider A, El Gueddari B (2001). Antibody responses to recombinant Epstein-Barr virus antigens in nasopharyngeal carcinoma patients: complementary test of ZEBRA protein and early antigens p54 and p138.. J Clin Microbiol.

[41] Dreyfus DH, Gelfand EW (1999). Comparative analysis of invertebrate Tc6 sequences that resemble the vertebrate V(D)J recombination signal sequences (RSS).. Mol Immunol.

[42] Oettinger MA (2004). How to keep V(D)J recombination under control.. Immunol Rev.

[43] Reddy YV, Perkins EJ, Ramsden DA (2006). Genomic instability due to V(D)J recombination-associated transposition.. Genes Dev.

[44] Miller G, El-Guindy A, Countryman J, Ye J, Gradoville L (2007). Lytic cycle switches of oncogenic human gammaherpesviruses(1).. Adv Cancer Res.

[45] Feschotte C, Pritham EJ (2007). DNA transposons and the evolution of eukaryotic genomes.. Annu Rev Genet.

[46] Feschotte C (2008). Transposable elements and the evolution of regulatory networks.. Nat Rev Genet.

[47] Pritham EJ, Putliwala T, Feschotte C (2007). Mavericks, a novel class of giant transposable elements widespread in eukaryotes and related to DNA viruses.. Gene.

[48] Hung CH, Liu ST (1999). Characterization of the Epstein-Barr virus BALF2 promoter.. J Gen Virol.

[49] Menetski JP, Gellert M (1990). V(D)J recombination activity in lymphoid cell lines is increased by agents that elevate cAMP.. Proc Natl Acad Sci U S A.

[50] Kolman JL, Taylor N, Gradoville L, Countryman J, Miller G (1996). Comparing transcriptional activation and autostimulation by ZEBRA and ZEBRA/c-Fos chimeras.. J Virol.

[51] Verkoczy L, Ait-Azzouzene D, Skog P, Martensson A, Lang J (2005). A role for nuclear factor kappa B/rel transcription factors in the regulation of the recombinase activator genes.. Immunity.

[52] Verkoczy LK, Martensson AS, Nemazee D (2004). The scope of receptor editing and its association with autoimmunity.. Curr Opin Immunol.

[53] Finkel TH, Kubo RT, Cambier JC (1991). T-cell development and transmembrane signaling: changing biological responses through an unchanging receptor.. Immunol Today.

[54] Gladow M, Uckert W, Blankenstein T (2004). Dual T cell receptor T cells with two defined specificities mediate tumor suppression via both receptors.. Eur J Immunol.

[55] Han S, Zheng B, Schatz DG, Spanopoulou E, Kelsoe G (1996). Neoteny in lymphocytes: Rag1 and Rag2 expression in germinal center B cells.. Science.

[56] Balmelle N, Zamarreno N, Krangel MS, Hernandez-Munain C (2004). Developmental activation of the TCR alpha enhancer requires functional collaboration among proteins bound inside and outside the core enhancer.. J Immunol.

[57] Hah C, Kim M, Kim K (2005). Induction of peripheral tolerance in dual TCR T cells: an evidence for non-dominant signaling by one TCR.. J Biochem Mol Biol.

[58] de Bruyn Kops A, Knipe DM (1994). Preexisting nuclear architecture defines the intranuclear location of herpesvirus DNA replication structures.. J Virol.

[59] Chen YM, Knipe DM (1996). A dominant mutant form of the herpes simplex virus ICP8 protein decreases viral late gene transcription.. Virology.

[60] Zhou C, Knipe DM (2002). Association of herpes simplex virus type 1 ICP8 and ICP27 proteins with cellular RNA polymerase II holoenzyme.. J Virol.

[61] Taylor TJ, Knipe DM (2003). C-terminal region of herpes simplex virus ICP8 protein needed for intranuclear localization.. Virology.

[62] Uprichard SL, Knipe DM (2003). Conformational changes in the herpes simplex virus ICP8 DNA-binding protein coincident with assembly in viral replication structures.. J Virol.

[63] Gellert M (2002). V(D)J recombination: RAG proteins, repair factors, and regulation.. Annu Rev Biochem.

[64] Jones JM, Gellert M, Yang W (2001). A Ku bridge over broken DNA.. Structure.

[65] Lu CP, Posey JE, Roth DB (2008). Understanding how the V(D)J recombinase catalyzes transesterification: distinctions between DNA cleavage and transposition.. Nucleic Acids Res.

[66] Lu CP, Sandoval H, Brandt VL, Rice PA, Roth DB (2006). Amino acid residues in Rag1 crucial for DNA hairpin formation.. Nat Struct Mol Biol.

[67] Dreyfus DH, Nagasawa M, Gelfand EW, Ghoda LY (2005). Modulation of p53 activity by IkappaBalpha: evidence suggesting a common phylogeny between NF-kappaB and p53 transcription factors.. BMC Immunol.

[68] Song JJ, Joshua-Tor L (2006). Argonaute and RNA–getting into the groove.. Curr Opin Struct Biol.

[69] Dreyfus DH, Jones JF, Gelfand EW (1999). Asymmetric DDE (D35E)-like sequences in the RAG proteins: implications for V(D)J recombination and retroviral pathogenesis.. Med Hypotheses.

